# The whole genome sequences and experimentally phased haplotypes of over 100 personal genomes

**DOI:** 10.1186/s13742-016-0148-z

**Published:** 2016-10-11

**Authors:** Qing Mao, Serban Ciotlos, Rebecca Yu Zhang, Madeleine P. Ball, Robert Chin, Paolo Carnevali, Nina Barua, Staci Nguyen, Misha R. Agarwal, Tom Clegg, Abram Connelly, Ward Vandewege, Alexander Wait Zaranek, Preston W. Estep, George M. Church, Radoje Drmanac, Brock A. Peters

**Affiliations:** 1 Complete Genomics, Inc., 2071 Stierlin Ct., Mountain View, CA 94043 USA; 2 Harvard Personal Genome Project, Harvard Medical School, NRB 238, 77 Avenue Louis Pasteur, Boston, MA 02115 USA; 3 PersonalGenomes.org, 423 Brookline Avenue, #323, Boston, MA 02215 USA; 4 Curoverse Inc., 212 Elm St, 3rd Floor, Somerville, MA 02144 USA; 5 BGI-Shenzhen, Shenzhen, 518083 China

**Keywords:** Complete genomics, Haplotypes, Long fragment read, LFR, Personal Genome Project, PGP, Whole genome sequencing

## Abstract

**Background:**

Since the completion of the Human Genome Project in 2003, it is estimated that more than 200,000 individual whole human genomes have been sequenced. A stunning accomplishment in such a short period of time. However, most of these were sequenced without experimental haplotype data and are therefore missing an important aspect of genome biology. In addition, much of the genomic data is not available to the public and lacks phenotypic information.

**Findings:**

As part of the Personal Genome Project, blood samples from 184 participants were collected and processed using Complete Genomics’ Long Fragment Read technology. Here, we present the experimental whole genome haplotyping and sequencing of these samples to an average read coverage depth of 100X. This is approximately three-fold higher than the read coverage applied to most whole human genome assemblies and ensures the highest quality results. Currently, 114 genomes from this dataset are freely available in the GigaDB repository and are associated with rich phenotypic data; the remaining 70 should be added in the near future as they are approved through the PGP data release process. For reproducibility analyses, 20 genomes were sequenced at least twice using independent LFR barcoded libraries. Seven genomes were also sequenced using Complete Genomics’ standard non-barcoded library process. In addition, we report 2.6 million high-quality, rare variants not previously identified in the Single Nucleotide Polymorphisms database or the 1000 Genomes Project Phase 3 data.

**Conclusions:**

These genomes represent a unique source of haplotype and phenotype data for the scientific community and should help to expand our understanding of human genome evolution and function.

**Electronic supplementary material:**

The online version of this article (doi:10.1186/s13742-016-0148-z) contains supplementary material, which is available to authorized users.

## Data description

### Utility of the dataset

In 2003, after 13 years of dedicated research and the public release of the reference human genome – a high-quality genome against which all later genome sequences would be compared – the Human Genome Project was officially declared complete, representing a stunning achievement for science and humanity. Since then, DNA sequencing technologies have rapidly improved and the cost of sequencing has outpaced Moore’s Law for almost the past 10 years [1]. More than 200,000 human genomes have been sequenced during this time but unfortunately, because an individual can be identified from their genome sequence, issues of anonymity have caused much of this data to sit in limited access databases. In addition, many datasets that are available to the public lack rich phenotypic data and so are of limited use. Finally, haplotype data has only been resolved for a small number of genomes and this important aspect of biology has been almost completely ignored in many studies. The genomic data published in this dataset represents the largest set of freely accessible whole individual genome sequences with phenotype and experimental haplotype information and should help to further our understanding of human biology.

### Sample collection

As part of the Personal Genome Project (PGP), blood samples from 184 participants were collected and processed using Complete Genomics’ Long Fragment Read (LFR) technology (Additional file 1). These participants gave full consent to have their genotypic and phenotypic data (Additional file 2) made freely and publicly available. Documents reviewed and signed by each participant can be found at [2]. Each PGP participant is given an opportunity to review their genome data and decide if they still wish to make it public. This process increases the time and uncertainty of data release and is the reason the complete set is currently not available. However, it is expected that the majority of these datasets will be released soon.

**Fig. 1:**
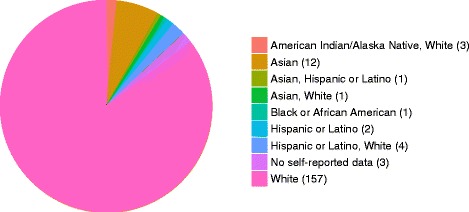
Self-reported participant ethnicity. As part of the Personal Genome Project sample acquisition process, participants were asked to report their ethnicity. The pie chart illustrates the proportion of samples from each ethnic group. Out of the 184 participants, more than 75 % reported themselves as White

A summary of the self-reported ethnicities of all 184 samples is displayed in Fig. 1. Certified phlebotomists collected blood samples at several events held across the United States. For each participant, blood samples were collected in two 5 ml EDTA tubes, labeled with anonymous identifiers, frozen within several hours and one of the tubes was shipped on dry ice to Complete Genomics (Mountain View, CA, USA).

### DNA isolation

Blood was thawed at Complete Genomics and 3 ml was used for DNA isolation with a RecoverEase dialysis kit (Agilent, Santa Clara, CA, USA). The remaining ~2 ml was frozen for later sample identification confirmation. High molecular weight genomic DNA was lightly fragmented by pipetting in and out of a P1000 pipette tip (Rainin Instruments LLC, Oakland, CA) 20 to 40 times. The final DNA concentration was measured using a Quant-iT™ Broad-Range dsDNA Assay Kit (Thermo Fisher Scientific, Waltham, MA). DNA samples were normalized to 10 ng/μl and stored at 4 °C.

### Library preparation and sequencing

Approximately 200 pg of genomic DNA (equivalent to the amount of DNA present in less than 30 cells) from each sample was used to make an LFR library [3] for sequencing. To make these libraries, about 5 ng of the genomic DNA was first denatured in 160 mM KOH and 1.0 mM EDTA in a total volume of 100 μl. After a 5-min incubation at 20 °C, 7 μl of this material was transferred to 26 μl of a 1 mM concentration of random DNA 8-mers in dH_2_O. After 2 min, an additional 32 μl of dH_2_O was added to the 8-mer solution resulting in a concentration of ~5.4 pg/μl of DNA and 400 μM of 8-mer in a final volume of 65 μl. 100 nl of this mixture was dispersed across a 384-well plate using a Mosquito® HTS Nanoliter Liquid Handler (TTP Labtech, Cambridge, UK) such that the final amount of DNA in each well was ~0.54 pg (~200 pg per LFR library). Multiple displacement amplification (MDA) [4] mix was added to a final volume of 1 μl to amplify the long genomic DNA fragments. After amplification, controlled random enzymatic (CoRE) fragmenting was performed and 300–1500 base-pair fragments were ligated to barcoded adapters unique to each well as previously described [3]. All libraries were sequenced using Complete Genomics’ nanoarray sequencing platform [5]. Both whole genome sequences and experimental phasing were obtained from each single “co-barcoded” [6] library generated from the LFR process. For seven samples, an additional 5 μg of genomic DNA was used for standard libraries. Standard libraries were processed and sequenced as previously described [5].

### Complete Genomics’ standard analysis pipeline and data formats

**Fig. 2:**
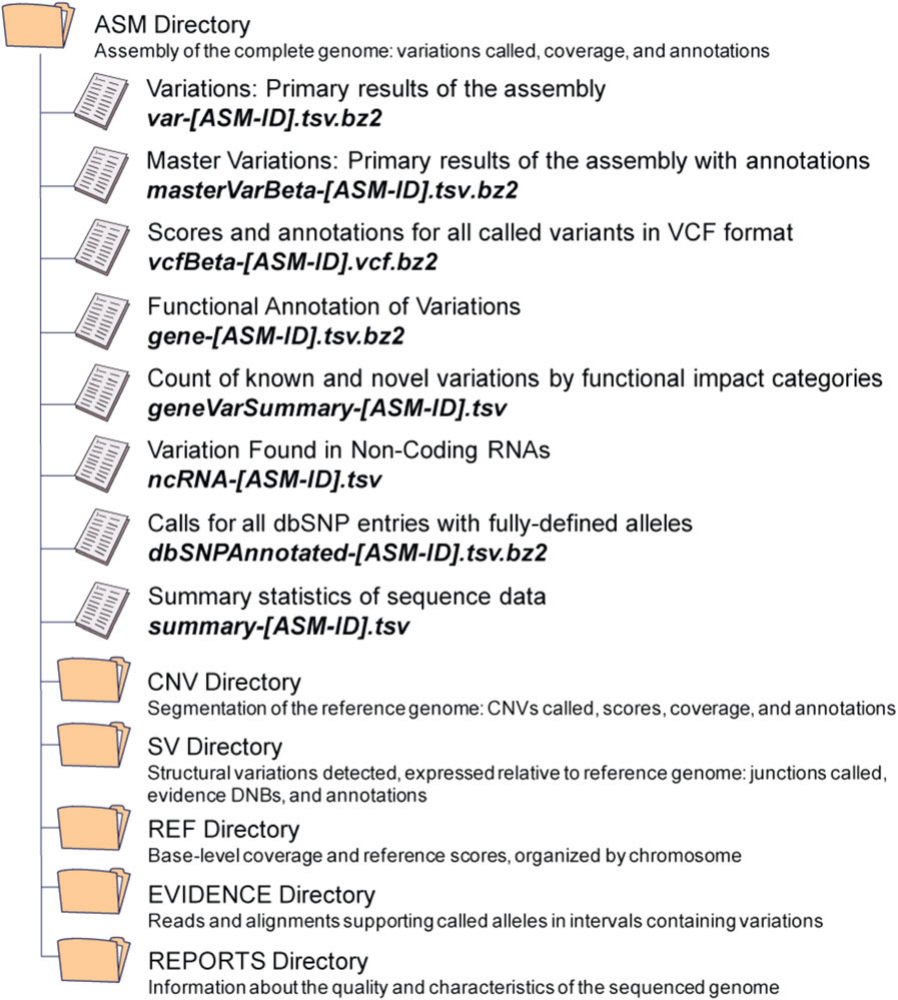
Data directory tree. The output from the Long Fragment Read (LFR) process consists of a series of files and folders. A complete description of everything contained within the Complete Genomics data package can be found in Additional file 3. *ASM* assembly, *CNV* copy number variations, *dbSNP* Single Nucleotide Polymorphisms database, *SV* structural variations, *VCF* variant cell format

The entire dataset from Complete Genomics consists of a series of files and directories covering various categories of whole genome analysis (Fig. 2). A complete description of all the files and methods used to generate this dataset is provided (Additional file 3 and is also available on the Complete Genomics’ website [7]).

### LFR analysis pipeline

**Fig. 3:**
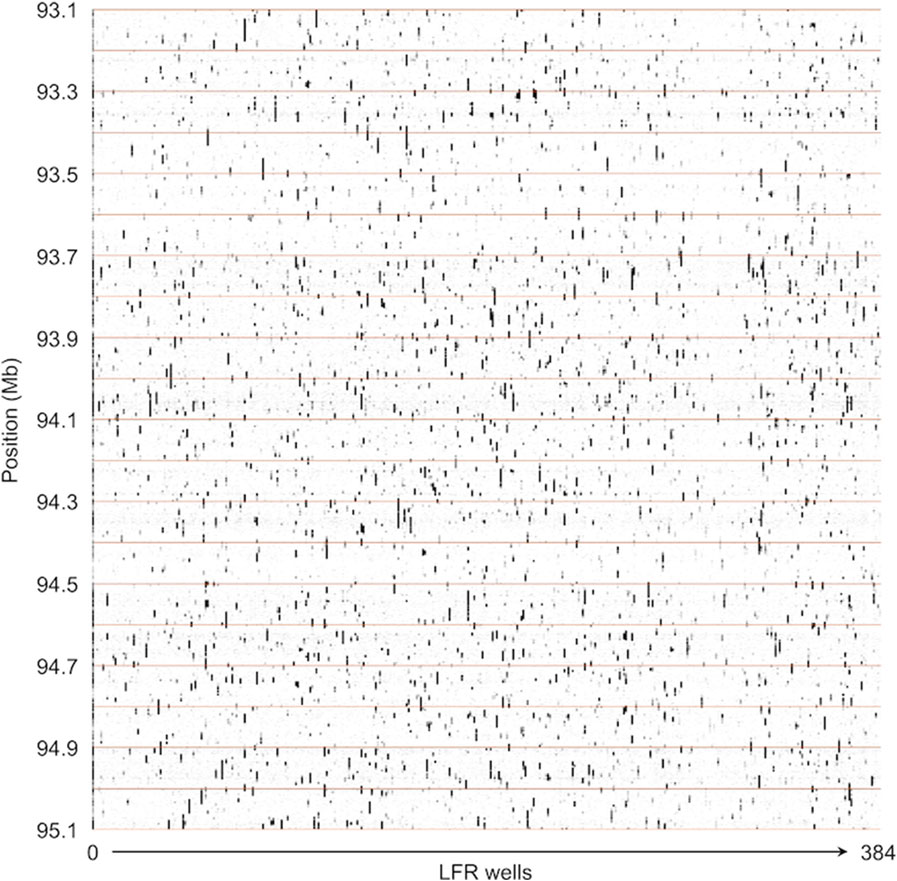
Coverage map. Mate-pair read coverage across all 384 wells of a Long Fragment Read (LFR) sample for the region on chromosome 14 from 93,100,000 to 95,100,000. From *left* to *right,* each column corresponds to one of the 384 wells, with the *leftmost column* corresponding to well 0 (this represents mate-pair reads for which the well was not called). The position in Mb along chromosome 14 is displayed on the vertical axis. Each red horizontal line corresponds to a 100 kb increment on chromosome 14. The gray scale encodes the number of mate-pairs mapped within each 1 kb bin. The fragments are clearly visible as vertical dark streaks in each column

Many of the steps for processing LFR are the same as those for standard libraries (described in Additional file 3). The processes specific to LFR are described in the following section. The first step unique to LFR is to create an unphased fragment map that gives, for each reference location, the list of wells that contribute to that location, but without knowledge of which wells contribute to each allele. To achieve this, a quick first mapping step is performed in which all mate-pair reads are mapped to the reference requiring an exact match of the entire mate-pair, with a mate gap consistent with the known mate gap range. Only mate-pair reads that have a unique exact mapping to the reference are used. We assign to the mapping a position equal to the average of the positions of the two arms. For each well, we maintain a mapping histogram that counts the number of mate-pairs that map in each 1 kb-wide bin. When this step is completed, we have a well coverage map, which gives the number of mappings in each 1 kb bin for each well (Fig. 3).

The unphased fragment map can be obtained from this coverage map using a simple algorithm that looks for streaks of several consecutive, well-populated 1 kb bins in the same well. This process allows for some missing coverage within a fragment due to non-unique regions of the genome that did not generate unique mappings. Fragments shorter than 10 kb are discarded. When this is done, we have a list of start and end positions for each fragment in each well – that is, an unphased fragment map. However, we do not yet know which wells, at a given reference position, contain the mate-pair reads that originated in each of the alleles.

We can use this information to construct a fragment-length distribution, which can be closely approximated by a decaying exponential with a decay length typically between 20 and 50 kb. This is consistent with simple DNA fragmentation models (e.g. breakage occurs with fixed probability at each location and in an uncorrelated fashion), which invariably predict exponential distributions of fragment lengths. A fragment coverage histogram – i.e. the distribution of the number of fragments covering each fragment location – can also be computed from this data. This is well-approximated by a Poisson distribution.

Next, the unphased fragment map is turned into a phased fragment map. This requires a second mapping step to be performed. Instead of mapping to the entire reference genome, we only map mate-pair reads in each well to the subset of the reference that is covered by the fragments in that well as determined by the unphased fragment map. In this second mapping step, only unique mappings with a single mismatch in each arm are allowed. All discordances between the reference and the mate-pair reads are tracked. Most of these are due to errors, but some of them are caused by heterozygous or homozygous single nucleotide polymorphisms (SNPs). To phase the fragments, a set of genomic positions that have strong support for two different base calls is collected. These are locations where a heterozygous SNP is highly likely to be present. Each of these “strong” heterozygous SNPs provides the two bases that correspond to the two alleles. From the second mapping step, we know which wells/fragments contain each of the two bases, and we can use this information to assign each fragment/well to an allele, meaning that we have turned the unphased fragment map into a phased fragment map. In an ideal situation, for a given SNP, all of the fragment/wells containing each of the two bases would be assigned to the same allele. However, because of errors and other artifacts, the well assignments to alleles will include contradictions. The algorithm that performs the allele assignment attempts to minimize the number of contradictions. If too many contradictions are present for a given fragment/well, the fragment is not assigned to any allele. In addition, in regions of low heterozygosity there are not enough SNPs to perform this assignment reliably. This can cause breaks in the allele assignment where it is not possible to relate the allele assignments at a given location to those some distance away. Each of the regions where it is possible to reliably assign the fragments to alleles without breaks is called a phased contig. Phased contigs are typically from a fraction of an Mb to a few Mbs in length.

With the phased fragment map available, local de novo assembly and variant calling can take place in a manner similar to the standard local de novo assembly described in Carnevali et al. [8] and Additional file 3. The only difference is that for most of the mate-pair reads that belong to a fragment that was phased, the allele that the mate-pair belongs to is known. If H is a hypothesis consisting of alleles A_0_ and A_1_, the standard formulation uses:

$$ \mathrm{P}\left(\mathrm{H}\Big|\mathrm{mate}-\mathrm{pair}\right) \propto \left(\mathrm{P}\left(\mathrm{mate}-\mathrm{pair}\Big|{\mathrm{A}}_0\right) + \mathrm{P}\left(\mathrm{mate}-\mathrm{pair}\Big|{\mathrm{A}}_1\right)\right)\ /\ 2 $$

That is, the mate-pair has equal prior probability of originating from each of the alleles. If the phased fragment map assigned the mate-pair to allele 0, the above becomes:

$$ \mathrm{P}\left(\mathrm{H}\Big|\mathrm{mate}-\mathrm{pair}\right) \propto \left(1 - \mu \right)\mathrm{P}\left(\mathrm{mate}-\mathrm{pair}\Big|{\mathrm{A}}_0\right) + \upmu \mathrm{P}\left(\mathrm{mate}-\mathrm{pair}\Big|{\mathrm{A}}_1\right)\Big) $$

Here, μ is small (we use μ = 0.01) and reflects the uncertainty of the fragment phasing process. As a result of this change, it is no longer the case that the probability of a hypothesis is invariant when the two alleles are swapped – in other words, hypothesis evaluation automatically takes phasing into account. The sequence optimization process works as in the standard formulation, taking into account this phasing-sensitive hypothesis probability at every step in the process. The benefit of this is two-fold: variant calls are intrinsically phased within each phased contig and the accuracy of variant calls is improved because of the additional information provided by the phased fragment map.

### LFR-specific files

**Fig. 4:**
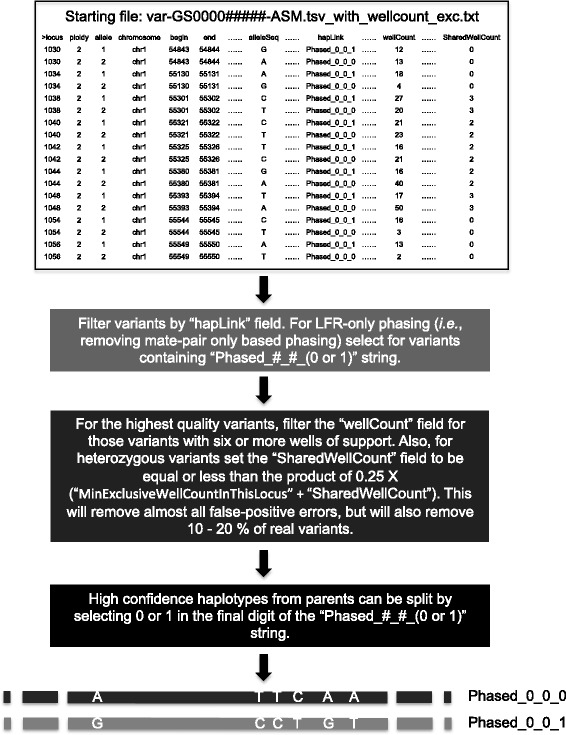
Haplotype extraction. Haplotypes can easily be retrieved from Long Fragment Read (LFR) samples starting from the variant file with file name format var-GS0000#####-ASM.tsv_with_wellcount_exc.txt. Following the steps provided in the figure will result in the highest quality haplotypes with an extremely low error rate, but with some loss of real variants. LFR haplotype performance using these filters has previously been described [3, 16]

Relative to the standard file formats (see Additional file 3), the data packages from LFR do not include a Mobile Element Insertion (MEI) directory or content in the Structural Variations (SV) directory. For an overview of the files and directories included, see Fig. 2. In addition, one of the fields in the variant file (hapLink) is modified and there are also six new fields, all of which are described in Table 1. How this file can be used with specific filters to isolate high-confidence haplotypes is shown in Fig. 4.

**Table 1:** Long Fragment Read-specific fields

Field	Description
hapLink	LFR phased variants have an ID with this pattern “Phased_#_#_#”, where # is an integer, the first two #s describe unique contigs, and the last # in the series is either 1 or 0 and represents the two possible haplotypes for each contig. All SNPs sharing the same “Phased_#_#_#” are from the same haplotype.
wellCount	Total number of LFR wells (out of 384) containing sequence reads calling the variant or reference allele. This metric is used to filter polymerase-induced false positive calls as it is unlikely that random polymerase errors will occur in several different wells. A complete explanation of this concept can be found in Peters et al. [16].
wellIDs	Contains the IDs of the specific wells from which reads calling the variant originate.
ecxclusiveWellCount	At each locus, this is the number of wells that have reads only calling the variant or the reference allele, not both; for true heterozygous variants, this number should be close to “WellCount”.
SharedWellCount	At each locus, this is the number of wells that contain reads calling both alleles; for true heterozygous variants, this should be low. A high number here suggests mapping errors and for homozygous variants, almost all of the well counts should be in this field.
MinExclusiveWell CountInThisLocus	At each locus, this is the minimum number of exclusive wells (non-shared well counts).
MaxExclusiveWell CountInThisLocus	At each locus, this is the maximum number of exclusive wells (non-shared well counts).

*LFR* Long Fragment Read, *SNP* single nucleotide polymorphism

### Genomic data quality

**Fig. 5:**
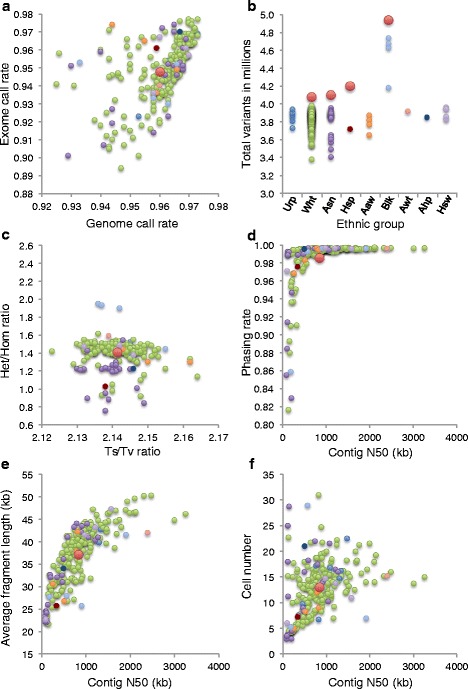
Genome quality metrics. Metrics from 225 individual genomic libraries (Additional file 1) from 184 Personal Genome Project (PGP) participants are plotted in each panel. Each dot represents a single genomic library from a PGP sample and is colored by ethnicity as follows: blue, Unreported (Urp); light green, White (Wht); purple, Asian (Asn); dark red, Hispanic or Latino (Hsp); light orange, American Indian/Alaska Native/White (Aaw); light blue, Black or African American (Blk); pink, Asian/White (Awt); dark blue, Asian/Hispanic or Latino (Asp); light purple, Hispanic or Latino/White (Hsw). The large red colored dot in panels **a**, **c**–**f** represents the average across the PGP data set. **a** The percent called across the genome is plotted on the x-axis and the percent called across the exome is plotted on the y-axis. **b** The total number of variant sites per genome is plotted on the y-axis and the ethnic group to which each sample was self-reported is plotted on the x-axis. Red colored dots represent the average number of single nucleotide polymorphisms (SNPs) in each population group as reported by the 1000 Genomes (1KG) project [9]. The ethnic groups in our study without a red dot lack a representative population in the 1KG data. **c** The heterozygous to homozygous SNP ratio (Het/Hom) is plotted on the y-axis and transition to transversion ratio (Ts/Tv) is plotted on the x-axis. **d** The SNP phasing rate is plotted on the y-axis and the N50 length of the assembled haplotype contigs in kilobases (kb) is plotted on the x-axis. **e** The average Long Fragment Read (LFR) fragment length is plotted on the y-axis and the N50 length of assembled haplotype contigs is plotted on the x-axis. Both values are in kb. **f** The number of cells-worth of genomic DNA was calculated based on assembled long fragment coverage and is plotted on the y-axis. The N50 length of the assembled haplotype contigs in kb is plotted on the x-axis

**Fig. 6:**
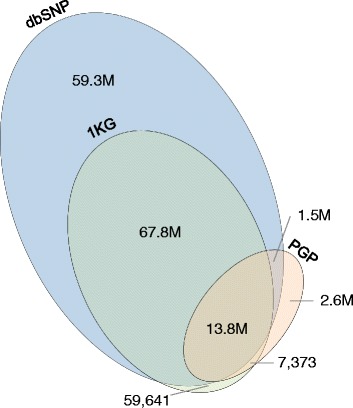
Venn diagram of the overlap between Personal Genome Project variants and those from the 1000 Genomes Project and the Single Nucleotide Polymorphisms database. Single nucleotide polymorphisms (SNPs) from all 225 Personal Genome Project (PGP) genomic libraries (Additional file 1) were filtered with the following criteria: 1) Each SNP must have a PASS in the “varFilter” field; this helps remove false-positive errors. 2) The variant call – and for heterozygous SNPs also the reference call – must have a “wellCount” of six or more; this removes most of the remaining false-positive errors. 3) For heterozygous SNPs, the “SharedWellCount” field is less than or equal to 0.25X (“MinExclusiveWellCountInThisLocus” + “SharedWellCount”); this removes potential mapping errors that result in an excess of wells for which both the reference and variant base is called. The combination of this set of filters has previously been shown [16] to remove the vast majority of false-positive errors and was chosen to create a set of very high confidence variants. This set was compared with variants in the 1000 Genomes (1KG, Phase 3) and the SNP database (dbSNP, Build 147) datasets. In total, more than 17 million SNPs were found in the PGP samples and these were compared with over 81 million and 142 million in 1KG and dbSNP, respectively. As expected, more than 85 % of SNPs found in the PGP samples were found in the 1KG and/or dbSNP datasets

Average call rates across the genomes and exomes of the PGP samples were high at 96 % and 94.5 % of the reference positions, respectively (Fig. 5a). The total number of variant sites per genome was similar to the 1000 Genomes (1KG) data [9] and showed similar variations between ethnic groups (Fig. 5b), although on average we reported several percent fewer variants. Heterozygous to homozygous SNP ratios (Het/Hom) and transition to transversion ratios (Ts/Tv) were near the expected values of 1.4–1.6 and 2.12–2.16, respectively, as based on prior large-scale sequencing studies [10, 11] (Fig. 5c). For most genomes, more than 98 % of heterozygous SNPs were placed into long haplotypes with an average N50 across PGP samples of 800 kb (Fig. 5d). Both a higher amount of starting DNA in each library and fragment length were found to correlate with longer N50 values (Fig. 5e, f). Comparison of SNP calls between standard and LFR libraries for the seven PGP samples analyzed by the two different methods showed high reproducibility with over 96 % overlap between the two data types (Additional file 4). In addition, over 85 % of the variant calls from all PGP genomes were found in the most recent data from 1KG (Phase 3) and the Single Nucleotide Polymorphisms database (dbSNP, Build 147)(Fig. 6). The remaining ~2.6 million variants not found in these datasets represent predominantly rare population and family-specific variants, false-positive errors, and de novo mutations. Based on previous literature [12–15] the expected number of de novo mutations per individual is typically less than 100. In total, only about 18,400 of these variants (100 × 184 unique participant samples), or about 0.7 %, can be attributed to de novo mutations. The filtering criteria we used (see Fig. 6) have previously been shown to remove the vast majority of false-positive errors [16]. Assuming roughly ten false-positive variants per genome library would yield a total of 2250 variants (10 × 225 genome libraries). This suggests that most of these ~2.6 million variants are real and rare in the population or are family-specific. This is similar to a recent study of high coverage, whole genome sequencing on more than 10,000 individuals [17].

**Fig. 7:**
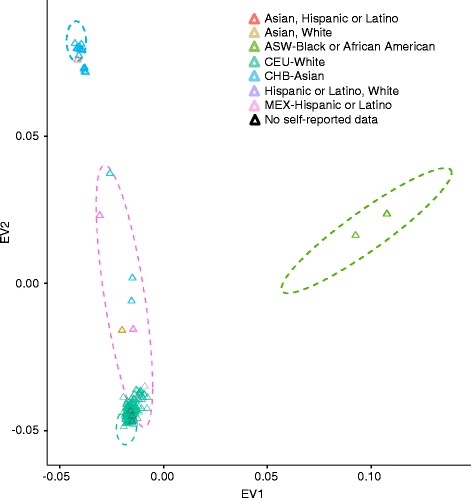
Principle component analysis. SNPRelate [21] was used to project 225 libraries (Additional file 1) from 184 Personal Genome Project (PGP) samples onto a principle component analysis using four different populations from the HapMap 3 project. Hierarchical clustering of this data using SNPRelate suggests that self-reported ethnicity for 182 of the 184 PGP samples matched the correct HapMap 3 ethnicity. The two PGP samples whose self-reported ethnicity did not cluster with the correct HapMap 3 ethnic group self-reported as Asian but their grandparents were of Indian and Sri Lankan ancestry. *ASW* African ancestry in Southwest USA, *CEU* Utah residents with Northern and Western European ancestry from the Centre de’Etude du Polymorphism Humain, Foundation Jean Dausset in Paris, France, *CHB* Han Chinese in Beijing, China, *EV* eigenvector, *MEX* Mexican ancestry in Los Angeles, California

**Fig. 8:**
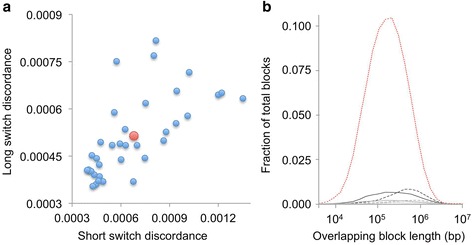
Pairwise comparisons of haplotype data between replicates. For each sample, replicate libraries were analyzed through pairwise comparisons. Single nucleotide polymorphisms (SNPs) were filtered with the same criteria as used in Fig. 6: 1) Each SNP must have a PASS in the “varFilter” field. 2) The “wellCount” field should be equal to six or greater for both variant and reference calls. 3) The “SharedWellCount” field must be less than or equal to 0.25X (“MinExclusiveWellCountInThisLocus” + “SharedWellCount”). In addition, overlapping blocks must contain at least ten SNPs and only pairwise comparisons between 1 million or more SNPs were analyzed. This final criterion reduced the number of pairwise comparisons to 35 and the number of participant samples to 12. This filter was applied to remove Long Fragment Read (LFR) libraries that were intentionally made with low coverage and thus have sparse haplotype coverage. Switch discordances were calculated by comparing the phase of heterozygous SNPs in completely overlapping blocks between replicate samples. **a** Short switch discordance rates were calculated by dividing the total number of discordant SNPs by the total number of phased SNPs in the compared blocks. Long switch discordance rates were calculated by dividing the total number of long switch events by the total number of phased SNPs in the compared blocks. Individual pairwise comparisons are represented by small blue dots on the plot and the average of all 35 comparisons is represented by the large red dot. **b** The fraction of total blocks with no errors (red dotted line), with one short (black solid line) or long (block dashed line) switch discordance, two to three short (solid grey line) or long (dashed grey line) switch discordances, or four or more short (solid light grey line) or long (dashed light grey line) were plotted against the block length in base pairs (bp). The vast majority of compared blocks (~86 %) have no discordances. Of those blocks that are discordant, very few have more than one short or long switch

To further confirm the quality of this dataset, LFR PGP assemblies were projected onto a principal component analysis (PCA) using four different populations from HapMap 3 (Fig. 7). In general, these results agreed closely with the self-reported ethnicities in Fig. 1. Furthermore, for 12 participant samples, replicate LFR libraries were compared for the purpose of determining reproducibility of haplotypes and measuring our phasing discordance rate. Thirty-five pairwise comparisons between replicates supported previous work [3], demonstrating that the LFR phasing was extremely reproducible with average short switch (a single SNP is out of phase) and long switch (a set of two or more SNPs switches haplotype) discordances of 0.00068 and 0.00051, respectively (Fig. 8a and Additional file 5). Importantly, the vast majority of long contigs did not contain a single discordant base between replicates and for those that did, most only contained a single short or long switch (Fig. 8b and Additional file 6). Taken together, these results suggest that this set of whole genome sequencing and haplotyping data is of a very high quality.

## Additional files

Additional file 1: PGP huIDs, ethnicities, and corresponding genome assembly IDs. (XLSX 20 kb)
Additional file 2: PGP participant phenotypes. (XLSX 48 kb)
Additional file 3: Standard Sequencing Service Data File Formats v2.5. (PDF 6212 kb)
Additional file 4: SNP call overlap between standard and LFR libraries. (XLSX 11 kb)
Additional file 5: Count of overlapping blocks, length, and SNPs for each pairwise haplotype comparison between replicates. (XLSX 22 kb)
Additional file 6: Count of overlapping blocks, length, and SNPs averaged from pairwise haplotype comparison between replicates. (XLSX 12 kb)

## Abbreviations

1KG: 1000 genomes
LFR: Long fragment read technology
PGP: Personal genome project
SNP: Single nucleotide polymorphism
